# The Enduring Strength of Biology and Gender: Care for Aging Parents Among Adult Children

**DOI:** 10.1093/geroni/igaa057.3340

**Published:** 2020-12-16

**Authors:** Sarah Patterson, Robert Schoeni, Vicki Freedman, Judith Seltzer

**Affiliations:** 1 University of Michigan, Ann Arbor, Michigan, United States; 2 University of California Los Angeles, Los Angeles, California, United States

## Abstract

Family complexity in the form of step-relationships are increasing across cohorts. Filial obligation, or the social norm that adult children should care for aging parents, are generally weaker in stepfamilies. Further, gender continues to be a main axis of stratification of who provides care within families. Taken together, we test whether biological versus step ties, the gender of the adult child, and the interaction between these two factors are associated with helping aging parents (ages 65 and older) with functional or health limitation based care needs. We use Round 5 (2015) of the National Health and Aging Trends Study. Results illustrate the enduring strength of both biological and gendered ties, with biological daughters being the most likely to help an aging parent, followed by biological sons, step-daughters, and lastly step-sons. This pattern holds even when we control for important characteristics of both the adult child and the care receiver. As families become more complex, these findings could mean that gaps in unmet care needs will emerge, especially for older adults who only have stepchildren.

